# Potential roles and mechanisms of bacterial peptidylarginine deiminase in dental biofilm mediated by *Porphyromonas gingivalis*

**DOI:** 10.1080/20002297.2025.2578893

**Published:** 2025-11-09

**Authors:** Yitong Chen, Jiale Lou, Ying Fang, Shibo Ying

**Affiliations:** a Savaid Stomatology School, Hangzhou Medical College, Hangzhou, Zhejiang, People's Republic of China; b School of Public Health, Hangzhou Medical College, Hangzhou, Zhejiang, People's Republic of China

**Keywords:** *Porphyromonas gingivalis*, porphyromonas peptidylarginine deiminase (PPAD), citrullination, dental biofilm, periodontitis

## Abstract

**Background:**

*Porphyromonas gingivalis*, a keystone oral pathogen, secretes the enzyme peptidylarginine deiminase (PPAD), which catalyzes protein citrullination and is implicated in both dental biofilm formation and the pathogenesis of systemic inflammatory diseases.

**Objective:**

This review aims to synthesize current knowledge on PPAD, with a specific focus on its mechanistic roles in oral biofilm dynamics and its potential contribution to the development of periodontitis and rheumatoid arthritis (RA).

**Design:**

A comprehensive literature search was conducted using the PubMed database up to August 2025, employing keywords including ‘PPAD’, ‘*Porphyromonas gingivalis*’, ‘citrullination’, ‘dental biofilm’, ‘periodontitis’, and ‘rheumatoid arthritis’.

**Results:**

PPAD contributes critically to biofilm pathogenicity by modulating microbial pH, citrullinating virulence factors, and facilitating polymicrobial interactions. It promotes bacterial adhesion, disrupts host immunity, and sustains local inflammation. Systemically, PPAD-generated citrullinated antigens may trigger autoimmune responses, potentially linking periodontitis to RA.

**Conclusion:**

PPAD represents a promising biomarker and therapeutic target for mitigating oral-systemic disease progression. Future research should prioritize elucidating its spatiotemporal regulation within biofilms and its immune-dysregulating effects to guide precision interventions.

## Introduction

Peptidylarginine deiminases (PADs) belong to a family of hydrolase enzymes that catalyze the post–translational conversion of peptidyl–arginine to peptidyl–citrulline–a process known as citrullination or deimination, which acts on carbon–nitrogen bonds outside of peptide linkages [1,2]. Human peptidylarginine deiminases (hPADs) constitute a conserved class of post–translational modification enzymes that regulate multiple physiological processes through arginine residue citrullination. First identified in the 1990s, five PAD isozymes (PAD1−4, 6) have been characterized to date [3]. These enzymes play indispensable roles in pivotal human biological pathways including epidermal differentiation, gene expression regulation, neutrophil extracellular trap (NET) formation, and tumorigenesis [1,2]. In addition to hPADs, the oral microbiome includes *Porphyromonas gingivalis* (*P. gingivalis*), a Gram–negative anaerobic bacterium that expresses a bacterial form of this enzyme, termed *Porphyromonas* peptidylarginine deiminase (PPAD) [4]. Homologs of PPAD are also present in other *Porphyromonas* species such as *P. gulae* and *P. loveana*, though these are derived from nonhuman hosts [5]. Although PPAD mediates citrullination reactions similar to those of hPADs, it exhibits several distinct biochemical characteristics. Notably, PPAD preferentially targets carboxy–terminal arginine residues and is also capable of deiminating free L–arginine, thereby generating unique antigenic profiles [6]. Moreover, unlike the calcium–dependent activity of hPADs, PPAD functions independently of Ca²⁺ [2,7]. Another key difference lies in inhibitor sensitivity: several compounds that effectively inhibit hPADs show no activity against PPAD [8]. Functionally, while hPADs predominantly operate within physiological systems, PPAD exhibits stronger intrinsic links to pathological mechanisms through its distinct catalytic properties in microbial communities.

*P. gingivalis* is a keystone pathogen in oral biofilms and is associated with gingivitis, periodontitis, and peri–implantitis [9]. It employs an array of proteolytic enzymes, secreted via the type IX secretion system (T9SS), to utilize host and matrix proteins for nutrient acquisition and virulence [10]. Among these, gingipains–arginine–specific (Rgp) and lysine–specific (Kgp) proteinases–and PPAD play central roles. Notably, Rgp generates C–terminal arginine residues that serve as optimal substrates for PPAD [7], suggesting a synergistic relationship between these enzymes. Though the precise mechanisms remain incompletely understood. PPAD has been implicated in the modulation and development of dental biofilms. Recent evidence also proposes the potential role of PPAD in systemic conditions such as rheumatoid arthritis (RA), possibly through citrullination–dependent pathways that promote autoantigen formation [11,12].

This review synthesizes current understanding of PPAD, with a specific emphasis on its mechanistic role in oral biofilm dynamics, and its contribution to the pathogenesis of periodontitis and RA. A comprehensive literature search of the PubMed database was conducted up to August 2025, utilizing keywords such as ‘PPAD’, ‘Porphyromonas gingivalis’, ‘citrullination’, ‘dental biofilm’, ‘periodontitis’, and ‘rheumatoid arthritis’. Through this review, we aim to synthesize current evidence and provide insights to guide the development of therapeutic and preventive strategies for associated diseases.

### Potential roles of PPAD in oral biofilms

**Figure 1.**
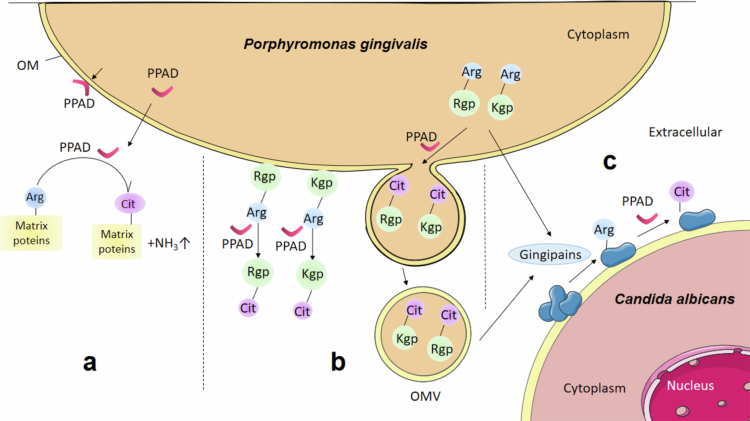
Overview of three roles of PPAD in the oral microbial community. Potential patterns of PPAD affecting oral biofilm formation and development include the following: (a) In oral biofilms, PPAD acts on arginine residues of target proteins to produce citrulline and the alkaline byproduct ammonia. (b) In mono–species biofilms, PPAD catalyzes the adhesion domains of the self–secreted proteases arginine–specific cysteine gingival proteinase R (Rgp) and lysine–specific cysteine gingival proteinase K (Kgp) and promotes their loading into outer membrane vesicles (OMVs). (c) In dual–species biofilm, gingipains including Rgp and Kgp may degrade cell surface proteins in *Candida albicans* and expose their arginine residues to PPAD. PPAD acts on these target proteins listed in Table S1, thus affecting the progression of the dual–species biofilms.

The oral biofilm known as dental plaque is a comprehensive ecological environment formed on the surface by interconnecting the microbial community and extracellular matrix. The adverse change in the natural balance of microcolonies may lead to oral diseases such as caries and periodontitis [13] *P. gingivalis* is known as an important oral microorganism which could disrupt the subtle balance of oral biofilms. PPAD is produced by this microorganism and considered as a novel virulence enzyme [14]. PPAD exists mainly in the following two forms: PPAD retained in the outer membrane (OM) of *P. gingivalis* cells and a soluble form associated with OMVs [15]. PPAD potentially modulates oral biofilm dynamics through three primary mechanisms: remodeling the pH microenvironment of microbial communities, post–translationally modifying endogenous virulence effectors, and mediating polymicrobial synergistic interactions. These three roles of PPAD are separately described below and also shown in Figure 1.

#### Role^1^: PPAD modulates microbial community pH dynamics

Oral biofilm pH exhibits spatial heterogeneity driven by differential microbial metabolism and structural architectures. Persistent acidic microenvironments (pH < 5.5) within microcolony interiors accelerate dental demineralization, while peripheral regions rapidly restore near–neutral pH through buffering mechanisms [16]. This dynamic gradient critically governs microbial succession patterns: early colonizers like Streptococcus mutans establish cariogenic niches via acidogenesis and extracellular polymeric substance synthesis [17], whereas late colonizers including *P. gingivalis* counteract acidity through PPAD–mediated enzymatic activity [18]. PPAD catalyzes the conversion of peptidyl–arginine to citrulline, releasing ammonia as a biochemical byproduct (Figure 1a) [6,19]. This ammonia production locally neutralizes acidic conditions, enabling *P. gingivalis* proliferation and supporting acid–sensitive commensals. Crucially, hyperactive PPAD variants, such as T2 isoform, demonstrate doubled citrullination capacity, significantly restructuring salivary microbiota composition [20]. Despite PPAD's established role in pH homeostasis, its synergistic interplay with acid–dependent enzymes and cross–kingdom signaling networks remains inadequately characterized, necessitating mechanistic investigations to guide periodontal therapeutics.

#### Role^2^: PPAD modifies endogenous virulence effectors

PPAD selectively citrullinates endogenous proteins in *P. gingivalis*, with substrate specificity demonstrating significant strain–dependent variation. Comparative exoproteome analyzes have identified distinct citrullination targets: six high–confidence modified proteins including virulence factors RgpA and Mfa1 fimbrilin in reference strains [21], while divergent clinical isolates exhibit citrullination predominantly in gingipain–derived adhesins (RgpA/Kgp) without fimbrial protein modification [22]. The expanding citrullinome encompasses 78 proteins harboring 161 modification sites within outer membrane vesicles (OMVs), indicating compartment–spanning enzymatic activity [23]. Notably, RgpA citrullination consistently occurs across reference strains (W83, ATCC 33277) and rheumatoid arthritis–associated isolates [21], whereas Mfa1 modification displays isolate–specific prevalence. Gingipains (RgpA, RgpB, Kgp)—T9SS–secreted proteases comprising prodomains, catalytic domains, and adhesin domains [24,25]—serve dual roles as PPAD substrates and functional modifiers (Figure 1b). Citrullination occurs principally within adhesin domains (e.g. Rgp27, Kgp39), reducing electrostatic potential through arginine charge elimination [22]. This post–translational remodeling alters subcellular trafficking: wild–type strains localize adhesins to cell surfaces and OMVs, while PPAD–deficient mutants accumulate them within biofilm matrices [22]. Such redistribution implies PPAD regulates OMV cargo loading and secretion, potentially governing biofilm structural dynamics. Functional consequences remain controversial: although citrullination reduces matrix accumulation in specific genetic backgrounds by weakening adhesin–matrix interactions [22], other strains (e.g. ATCC 33277) show unaltered biofilm architecture despite PPAD activity [26]. PPAD critically governs OMV biogenesis through its enzymatic activity, enabling efficient packaging and delivery of concentrated virulence payloads–including gingipains and active PPAD–which drive surface translocation and microenvironment remodeling. This citrullination–dependent process establishes a self–amplifying virulence circuit where PPAD simultaneously modifies endogenous effectors, reprograms arginine metabolism, and regulates vesicular transport, collectively enhancing tissue colonization while subverting host defenses through protease–loaded OMVs [27]. These contradictions highlight context–dependent functionality influenced by growth conditions and genetic determinants, emphasizing the need for standardized models to resolve PPAD's mechanistic contributions to biofilm pathogenesis.

#### *Role^3^*: PPAD mediates polymicrobial biofilm synergy​​​​​

PPAD critically governs interspecies partnerships in dual–species biofilms, particularly between *P. gingivalis* and the metabolically versatile fungus *Candida albicans* (Figure 1c). Through mitochondrial oxygen scavenging, *C. albicans* creates anaerobic microniches enabling obligate anaerobes like *P. gingivalis* to proliferate under aerobic conditions [28–30]. This syntrophic interaction significantly enhances bacterial viability during co–culture. PPAD activity specifically regulates time–dependent adhesion dynamics: while initial 3 hours attachment shows negligible differences between PPAD–expressing and deficient strains, wild–type adhesion to fungal surfaces increases more than 2-fold by 24 hours [31], confirming PPAD's essential role in stabilizing mature biofilm consortia. Surface proteomics of co–cultured *C. albicans* reveals nine PPAD–modified targets: glycolytic enzymes (enolase 1, hexokinase−2, phosphoglycerate kinase), metabolic regulators (alcohol dehydrogenase 1), structural components (mannoprotein MP65, *β*-glucanase), and stress–response effectors (Ssb1, pH–regulated antigen) [31]. These strategic modifications potentially reconfigure fungal–bacterial interfaces by disrupting host–pathogen recognition epitopes, altering metabolic cross–feeding dynamics and signaling networks. Given *C. albicans*' role in multispecies biofilm pathogenicity [32], PPAD–mediated citrullination represents an evolved virulence adaptation enhancing bacterial exploitation of fungal partners. PPAD orchestrates biofilm structural integrity through quorum sensing–mediated interactions with *Actinomyces* and *Tannerella* species, enhancing coaggregation stability [33]. Citrullination of outer membrane proteins further reinforces interbacterial adhesion, functioning as molecular cement that fortifies biofilm architecture [31,34,35]. These biochemical modifications concomitantly elevate antimicrobial resistance through matrix densification and altered permeability. Future studies should delineate how individual modified proteins mediate interspecies adherence thresholds and determine whether citrullination modulates antifungal resistance in polymicrobial contexts, thereby informing novel therapeutics targeting dysbiotic biofilm communities in systemic diseases.

### PPAD and periodontitis

Periodontitis, a chronic immune–inflammatory disease characterized by the progressive destruction of tooth–supporting tissues, is strongly associated with the keystone pathogen *P. gingivalis* residing in subgingival plaque [36]. PPAD produced by this bacterium plays a central role in disease pathogenesis through multiple synergistic mechanisms. It enhances bacterial adhesion to and invasion of host gingival cells, suppresses neutrophil phagocytosis, and inhibits neutrophil apoptosis, thereby prolonging inflammatory responses [37]. The secreted PPAD may neutralize innate immune defenses by enabling bacterial escape from NETs through citrullination of histone H3, as well as by inactivating the antibacterial lysozyme–derived peptide LP9 via citrullination [38]. Furthermore, PPAD stimulates the release of proinflammatory cytokines including tumor necrosis factor *α* (TNF-*α*) and interleukin−6 (IL−6), while also activating osteoclast–mediated bone resorption through the prostaglandin E2 pathway [39,40]. Recent studies reveal significant genetic heterogeneity in PPAD across different stages of periodontitis, with specific polymorphic variants upregulating TNF-*α* and IL−6 expression, thereby exacerbating disease severity [41]. The clinical relevance of PPAD is further highlighted by its strong correlation with key periodontal parameters. The clinical relevance of PPAD is further underscored by its correlation with key periodontal parameters; future investigations should prioritize elucidating the precise role of PPAD as a predictive biomarker and its potential as a therapeutic target to improve clinical management of periodontitis.

### PPAD and rheumatoid arthritis

Rheumatoid arthritis (RA) is a chronic autoimmune and inflammatory disorder characterized by persistent synovitis, joint swelling, pain, stiffness, and progressive tissue damage [42]. A hallmark of RA is the presence of autoantibodies, particularly rheumatoid factor and anti–citrullinated protein antibodies (ACPAs), which serve as well–established serological markers and contribute to disease pathogenesis [43,44]. Emerging evidence implicates oral pathogens, especially *P. gingivalis,* in the initiation and perpetuation of autoimmunity in RA. Transient bacteremia induced by routine activities such as tooth brushing may facilitate the systemic dissemination of citrullinated peptides or proteins derived from the oral cavity [45]. These externally derived citrullinated antigens can break immune tolerance and promote ACPA production, ultimately triggering autoimmune attacks characteristic of RA. Although *P. gulae* also expresses PPAD, no significant correlation has been found between its presence or its specific anti–citrullinated peptide antibodies and clinical markers of RA, underscoring the unique role of *P. gingivalis* as a primary exogenous source of pathogenic citrullination [46]. Beyond adaptive immunity, *P. gingivalis* employs multiple virulence mechanisms involving PPAD. Recent studies demonstrate that PPAD, in concert with major fimbriae FimA, activates Toll–like receptor 2 (TLR2), initiating potent proinflammatory responses and stimulating prostaglandin E₂ synthesis in gingival fibroblasts [47]. Type I fimbriae exhibit the most robust proinflammatory activity among various fimbrial types [48], and TLR2 signaling pathways are now considered promising therapeutic targets for RA treatment [42]. Importantly, epidemiological studies across diverse populations support the clinical relevance of these mechanisms. Indonesian subjects with elevated IgA ACPA levels in gingival crevicular fluid showed significantly higher abundance of *P. gingivalis*, linking periodontopathic bacteria to local autoimmune responses [49]. Similarly, in Colombian cohorts, elevated serum levels of anti–RgpA and anti–RgpA/PPAD antibodies were identified in RA patients, suggesting their potential utility as diagnostic biomarkers [50]. Furthermore, RA may predispose individuals to secondary infections; *P. gingivalis* OMVs promote aggregation and neutrophil–mediated internalization of Staphylococcus aureus, facilitating its dissemination into the bloodstream and increasing the risk of bacteremia [51]. Particularly, PPAD–mediated citrullination drives OMV biogenesis in *P. gingivalis*, packaging bioactive virulence cargoes that disseminate into systemic circulation, thereby establishing a mechanistic conduit for the established correlation between periodontitis and autoimmune conditions like RA through systemic propagation of citrullinated antigens and inflammatory mediators [27]. Given the central role of PPAD in protein citrullination and immune activation, it represents a compelling therapeutic target for RA and related autoimmune conditions.

### Conclusions and prospects

PPAD has been established as a central virulence regulator in periodontal pathogenesis, operating through three core mechanisms: modulation of microbial community pH, post–translational modification of endogenous effectors, and mediation of polymicrobial synergy. It amplifies periodontitis severity by enhancing bacterial adhesion, disrupting neutrophil function, and sustaining inflammation, while systemically disseminated PPAD–generated citrullinated antigens contribute to autoimmune pathways such as rheumatoid arthritis. These insights nominate PPAD as a promising therapeutic target. Future efforts should prioritize deciphering PPAD’s context–dependent expression and activity within dynamic biofilms, and developing combinatorial interventions that simultaneously inhibit PPAD and restore microbiome balance. Embracing PPAD’s dual role as a molecular target and ecological modulator will advance precision therapeutics for periodontitis and PPAD–linked systemic diseases.

## Supplementary Material

Supplementary Material TableS1
